# Supplementary material for: SREBP-dependent lipidomic reprogramming as a broad-spectrum antiviral target
Source: Nat Commun. 2019 Jan 10;10:120. doi: 10.1038/s41467-018-08015-x (PMC6328544; doi:10.1038/s41467-018-08015-x)
Supplement: Supplementary file 1 — Supplementary Information [file 41467_2018_8015_MOESM1_ESM.pdf]

Supplementary information

SREBP-dependent lipidomic reprogramming as a broad-spectrum antiviral target

Yuan *et al.*

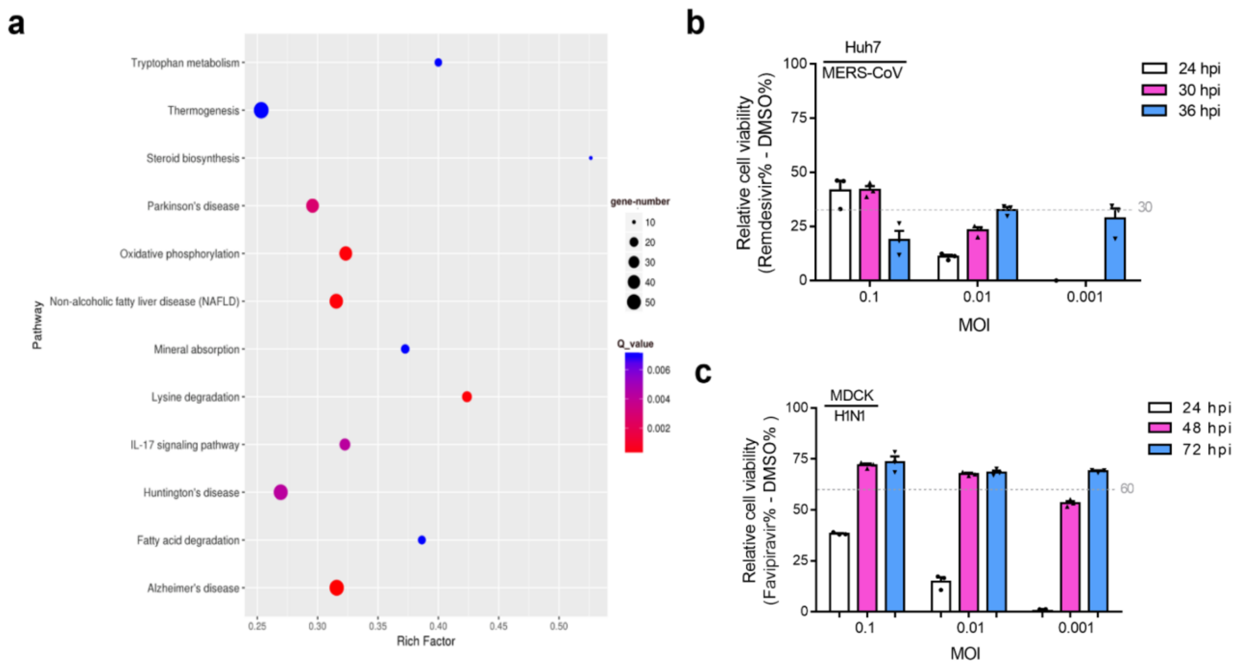

Supplementary Figure 1. Transcriptomic analysis and lipid library screening. (a) KEGG pathway analysis of differentially expressed genes (DEGs) when comparing the MERS-CoV infected and non-infected Calu-3 cells (2 MOI, 24hpi). Advanced bubble chart shows enrichment of DEGs in signaling pathways. Size and color of the bubble represent amount of DEGs enriched in pathway and enrichment significance, respectively. Pathways with Q_value (*p* value) < 0.01 are presented. Rich factor=amount of DEGs enriched in the pathway/amount of all genes in background gene set. (b and c) Optimization of MOI conditions and time-points for screening of anti-MERS-CoV and anti-H1N1 inhibitors as indicated. Remdesivir (5µM) and Favipiravir (50µg/ml) were used as a positive control for MERS-CoV and H1N1, respectively. Results are normalized by the cell-viability of mock-infected cells. The experiments were performed in triplicate and repeated twice for confirmation. Data are presented as mean+s.d.

a

Cytotoxicity of AM580

Cell line	CC_{50} (μM) MTT assay	CC_{50} (μM) CellTiter-Glo assay
Huh7	118.1 ± 1.1	106.2 ± 3.1
HEp-2	132.6± 1.2	152.6± 6.5
RD	127.6 ± 3.2	117.4 ± 2.1
HEK293T	115.7 ± 3.2	192.1 ± 6.3
Vero	126.7 ± 1.7	135.4 ± 2.9
MDCK	212.8 ± 12.3	142.8 ± 4.1

b

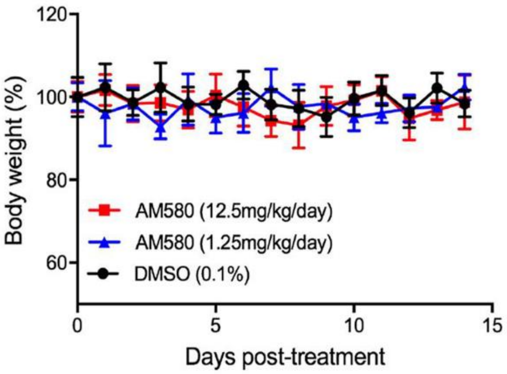

c

Virus family	Virus	IC_{50} (μM)	SI (CC_{50}/IC_{50})
Coronaviridae	MERS-CoV (HCoV-EMC/2012)	0.25 ± 0.03	507
	SARS-CoV (GZ50)	1.11 ± 0.83	114
Orthomyxoviridae	Influenza A (H1N1)pdm09	1.34 ± 1.07	159
Picornaviridae	EV-A71 (SZ/HK05)	2.19 ± 1.35	58
Flaviviridae	ZIKV (PRVABC59)	5.70 ± 1.08	22
Adenoviridae	Human AdV 5 (clinical isolate)	1.51 ± 0.37	88

Supplementary Figure 2. Cytotoxicity and antiviral activity of AM580. (a) CC_{50} of AM580 in different cell lines determined by measuring either the cellular NAD(P)H-dependent cellular oxidoreductase enzymes (MTT assay) or ATP activity (CellTiter-Glo assay), respectively. (b) *In vivo* toxicity of AM580 to DDP4 Tg mice (n=4) were checked by intraperitoneal (i.p.) injection of AM580 or DMSO (0.1%) for 7 days. Shown is the body weight change of each group recorded for 14 days. The result is show as mean body weight each day ± s.d. (c) IC_{50} of AM580 against different viruses determined by PRA except AdV5 by $TCID_{50}$ assay. Selectivity index (CC_{50}/IC_{50}) of AM580 antiviral treatment against different viruses.

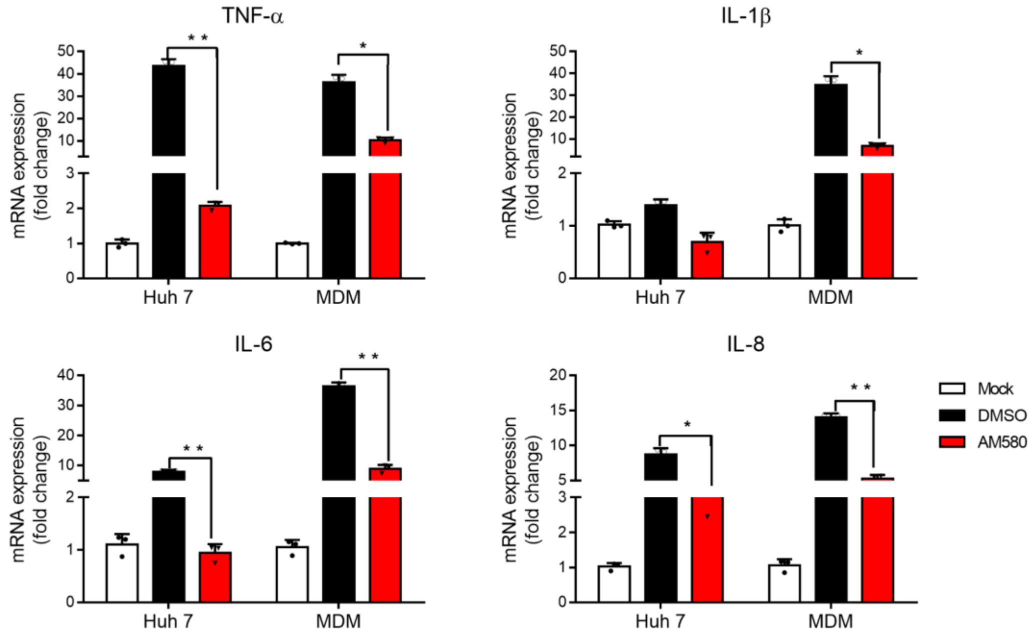

Supplementary Figure 3. Gene expression analyses of MERS-CoV-infected cells with or without AM580 treatment. AM580 reduced the pro-inflammatory cytokine up-regulation in Huh7 cells and Monocyte-derived macrophages (MDMs) infected by 1 MOI of MERS-CoV at 12hpi. Shown are representative cytokine markers analyzed by RT-qPCR, respectively. The differences between MERS-CoV treated by DMSO and by AM580 were statistically analyzed with one-way ANOVA. *p< 0.05, **p<0.01. The experiments were performed in triplicate and repeated twice for confirmation. Data are presented as mean ± s.d.

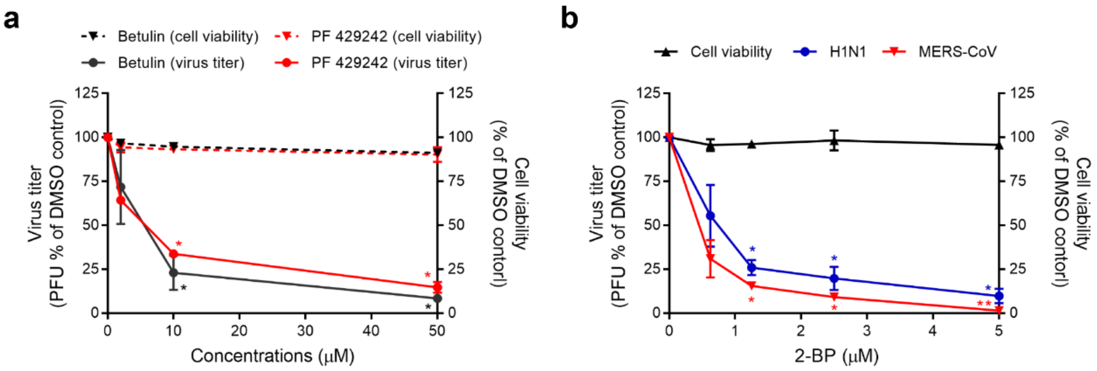

Supplementary Figure 4. Suppression of SREBP-associated pathways reduced virus replication. (a) n-SREBP processing inhibitors showed anti-MERS-CoV activity. PF 429242 is a site 1 protease (S1P) inhibitor that prevents the cleavage of pre-SREBPs to n-SREBPs, while betulin stimulate the association of SREBP cleavage activating protein (SCAP) and Insig so that the SREBP maturation is blocked. Huh7 cells were infected with MERS-CoV and treated with compounds with indicated concentrations or vehicle control (0.1% DMSO). Viral titer in the supernatant was analyzed by plaque assay at 24hpi (b) 2-BP, a protein palmitoylation inhibitor, reduced both MERS-CoV and H1N1 virus replication. MDCK cells were infected by H1N1 at 0.001 MOI and treated with 2-BP at nontoxic concentrations as indicated. After 24 h, viral titer in the supernatant was analyzed by plaque assay. 2-BP also reduced MERS-CoV replication in Huh7 cells in a dose-dependent manner. Cell viability under the treatment of different compounds were evaluated by MTT assays for 24h. One-way ANOVA was used to compare the inhibitor treated and DMSO groups. $**p<0.01$, $*p<0.05$. Data represents mean± s.d. from two independent experiments with triplicates.

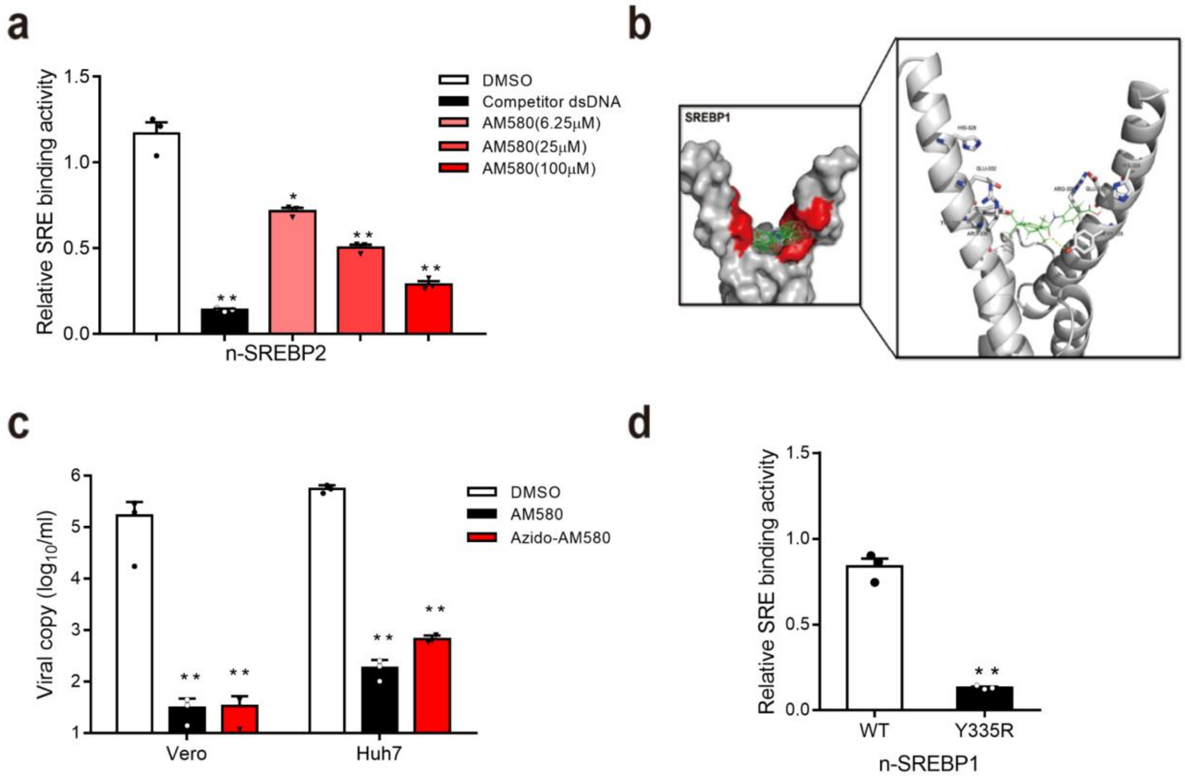

Supplementary Figure 5. AM580 disrupted n-SREBP and SRE interaction. (a) n-SREBP2 DNA binding activity assay showed that AM580 inhibited n-SREBP2 binding to its SRE in a dose-dependent manner. One-way ANOVA was used to compare AM580-treated group with the DMSO-treated control. Competitor dsDNA was used as a positive control inhibitor. (b) Docking analysis predicts the key amino acid residues of SREBP1 that interact with AM580. DNA-binding domain of SREBP1 is shown as a homodimer, where AM580 occupies the SRE recognition site through contacting with His328, Glu332, Tyr335 and Arg336. Dotted line indicates hydrogen bond is formed between AM580 and residue Tyr335. (c) The chemically modified compound of AM580, azido-AM580, displayed similar antiviral potency in MERS-CoV infected Vero and Huh7 cells. One-way ANOVA was used to compare the supernatant viral load in azido-AM580-treated or AM580-treated group with the DMSO-control group. (d) Tyr335 was critical for n-SREBP1 binding activity. WT n-SREBP1 and its mutant plasmid construct Y335R were transfected in HEK293T cells for 48h, followed by nuclear extraction and measurement of DNA binding intensity using the SREBP1 transcription factor assay kit. Difference between WT and Y335R was analyzed with the Student's t test. **p<0.01, *p<0.05. The experiments were carried out in triplicate and repeated twice for confirmation. Data are presented as mean ± s.d.

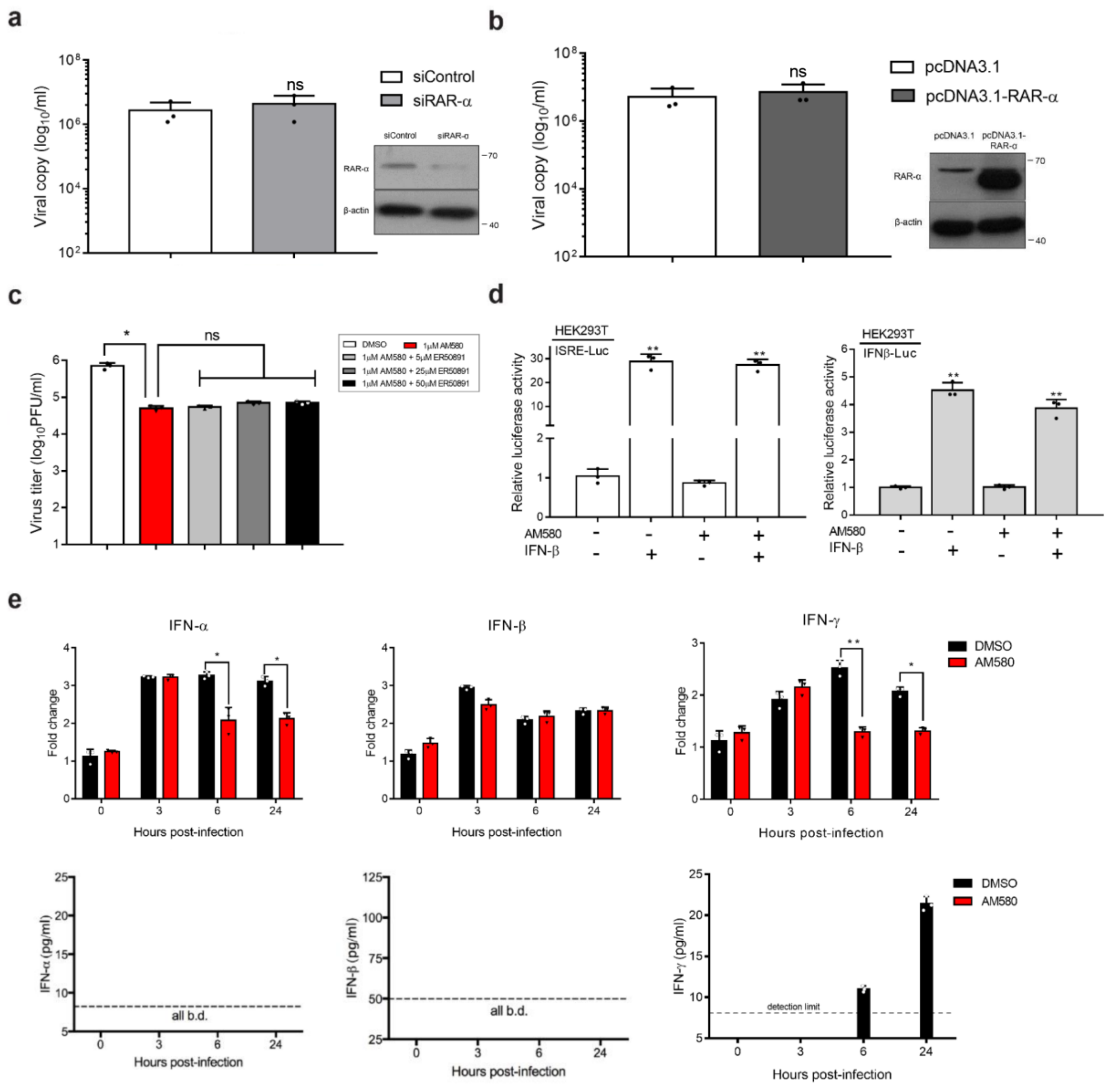

Supplementary Figure 6. Antiviral activity of AM580 was not dependent on RAR-α-signaling or innate antiviral response. (a and b) Huh7 cells were transfected with RAR-α-targeted siRNA (a) or RAR-α overexpression plasmid (b) for 24h, followed by MERS-CoV infection (0.01 MOI) for another 24h. Viral load in the cell culture supernatants are shown. Efficiency of siRNA knock-down or overexpression of RAR-α was detected by western blot using RAR-α specific antibody and β-actin. Differences between groups were compared by Student's t-test. ns indicates $p>0.05$. (c) Combined use of RAR-α antagonist ER50891 and AM580 did not reduce the antiviral activity of AM580. *$p<0.05$ when compared with DMSO control by one-way ANOVA. (d) AM580 was not related to host innate immune response activation and did not function as a signaling amplifier. Dual-luciferase assays were performed to evaluate the expression of reporter genes as indicated. IFN-β (100units/ml) was used as a positive control for the measurement of IFNβ-Luc and ISRE-Luc activities. Student's t-test was performed comparing with the mock-treated group. **$p<0.01$. (e) MDMs were infected by MERS-CoV for 2h (2 MOI), followed by PBS wash and replaced with fresh medium with or without AM580. Cell lysate were collected for real-time PCR at the indicated time points post-infection, in parallel, supernatant was harvested for ELISA detection of IFNα/β/γ, respectively. The qPCR results were normalized by human house-keeping gene GAPDH, fold change indicates fold change to the mock-infected cells. The dotted lines indicate detection limitation, which are 8pg/ml for IFNα and IFNγ, while 50pg/ml for IFNβ, respectively. The concentration of AM580 (20μM) and DMSO (0.1%) were consistent among the luciferase, IFN qPCR and ELISA assays. All b.d.: all below detection limit. **$p<0.01$ and * $p<0.05$ when compared by one-way ANOVA. The experiments were carried out in triplicate and repeated twice for confirmation. Data are presented as mean ± s.d.

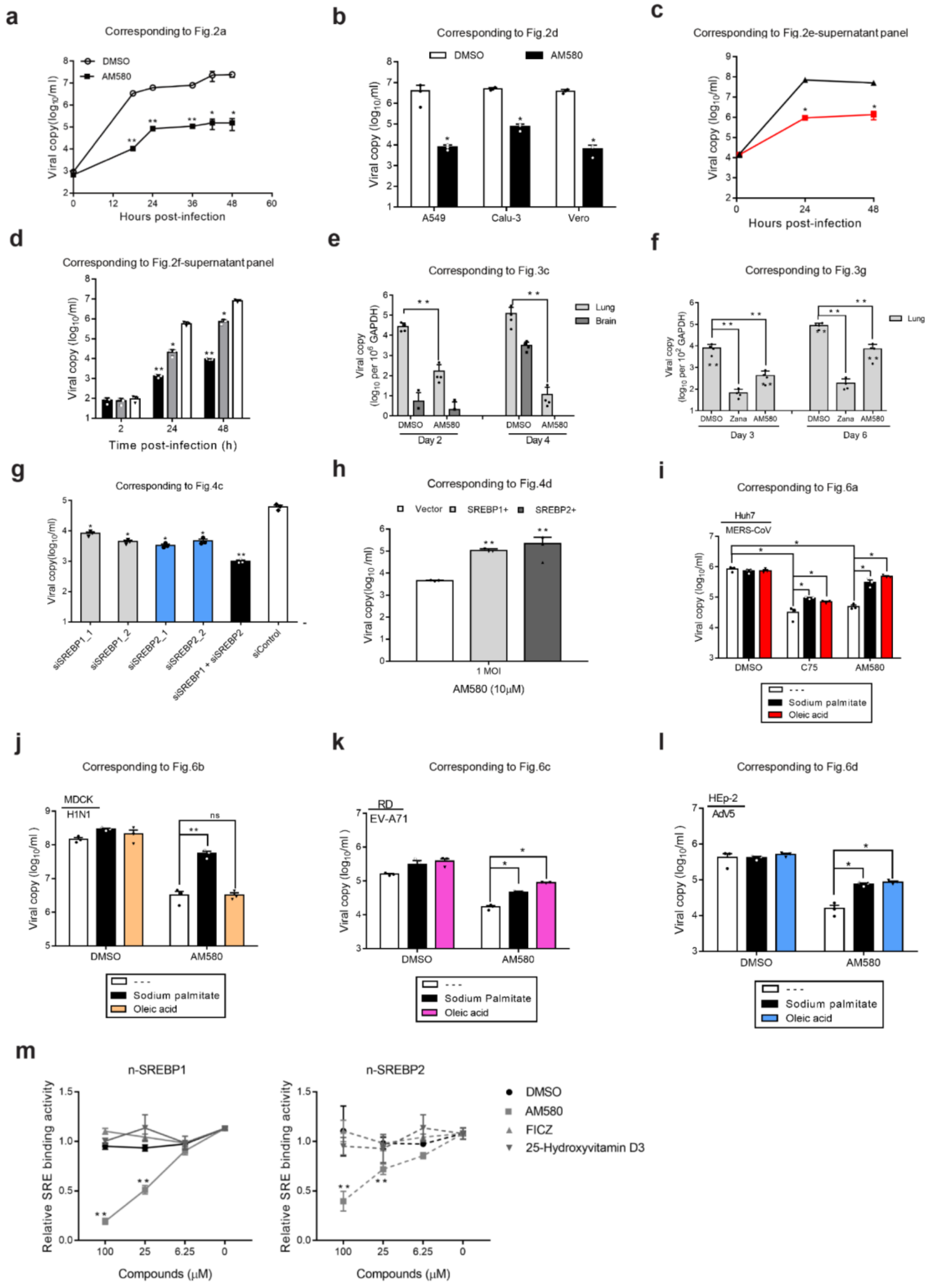

Supplementary Figure 7. Corresponding results of the main figures. (a-l) RT-qPCR results corresponding to the main figures as indicated. Number of experimental replicates and statistical tests are equivalent as that of the main figures. (m) The compounds FICZ and 25-Hydroxyvitamin D3 did not inhibit SRE binding activity to n-SREBP1 or n-SREBP2. Details of the methodology was described in the legends of main Figure 5a and 5b.

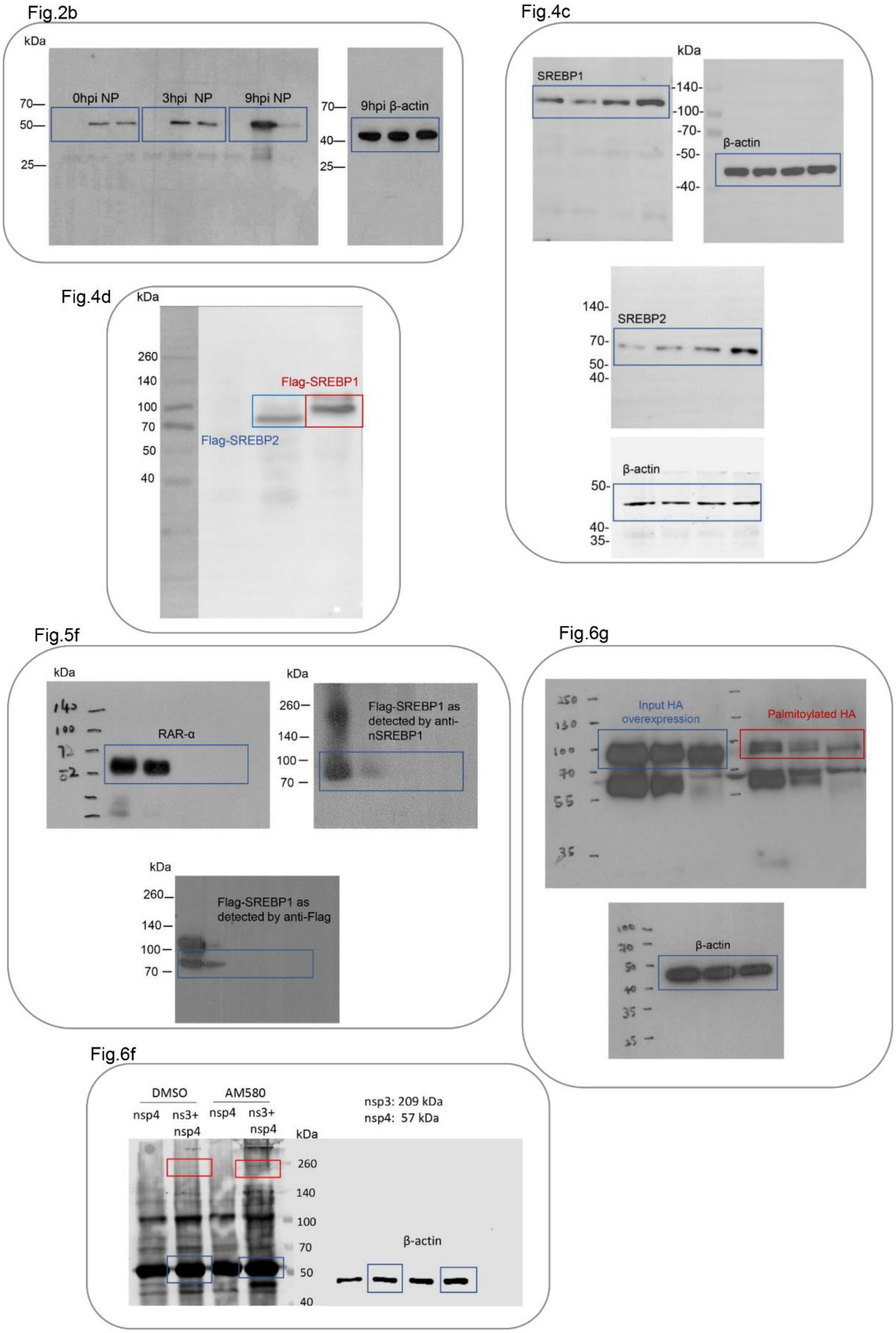

Supplementary Figure 8. Full blots.

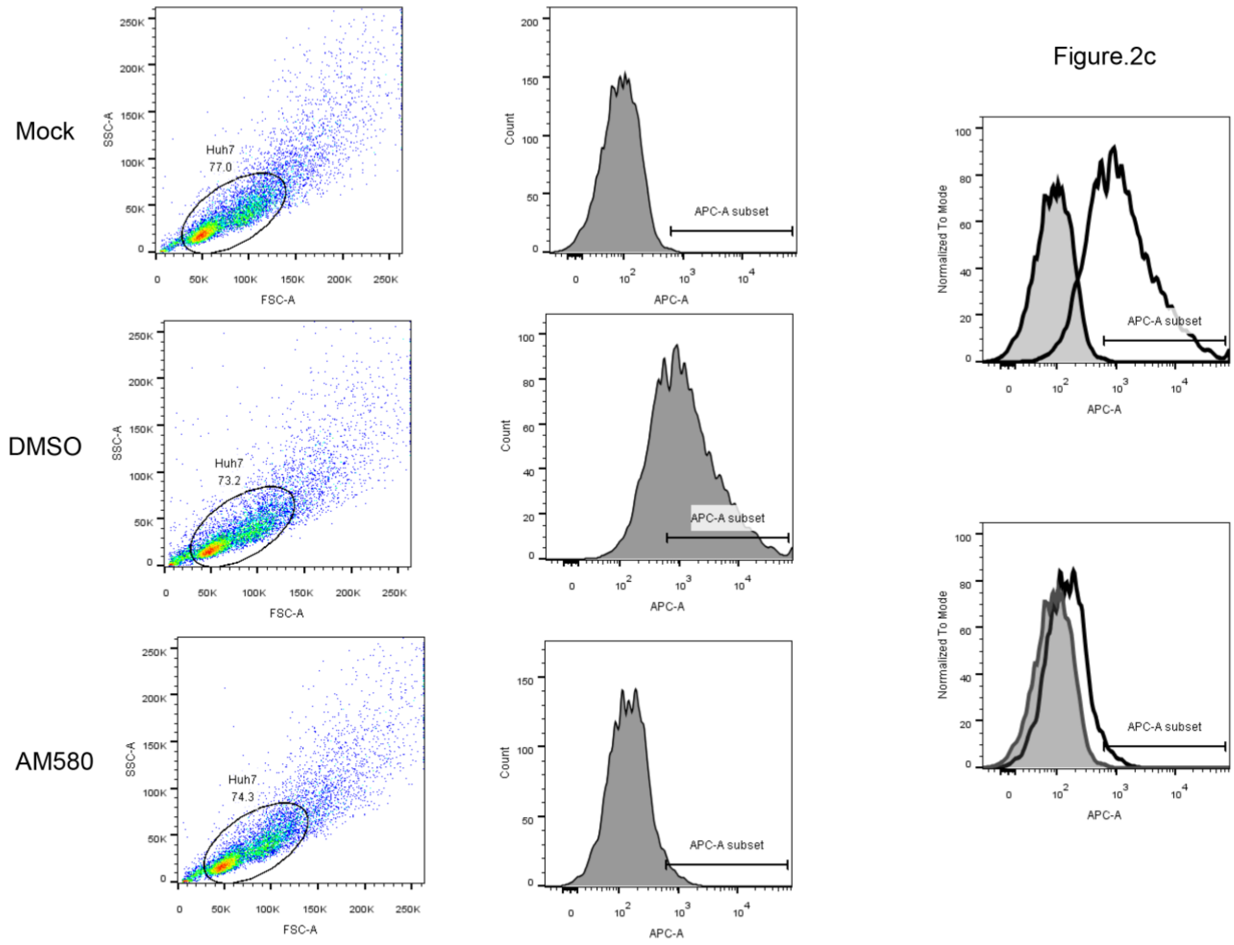

Supplementary Figure 9. Gating strategy for Figure 2c.

Supplementary Table 1. Antiviral activity and biological function of AM580 analogs.

Compound	Structure	IC_{50} (nM)*	Biological functions
AM580	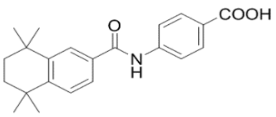	250 ± 32	RARα agonist
Tamibarotene	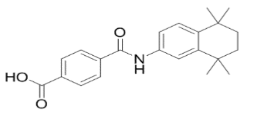	320 ± 26	RARα/β agonist
Bexarotene	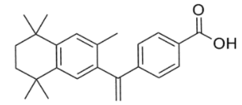	875 ± 15	Retinoid X receptor (RXR) agonist
LG100268	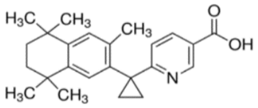	540 ± 14	unknown
BMS-195614	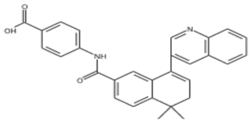	980 ± 34	unknown
BMS-189453	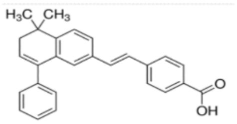	1230 ± 85	unknown
S61752	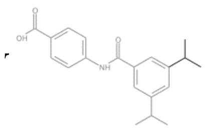	1180 ± 56	unknown
Palovarotene	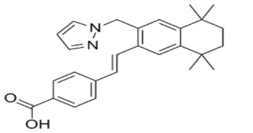	No activity	RARγ agonist
TTNPB	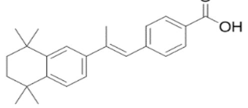	No activity	RARα/β/γ agonist
	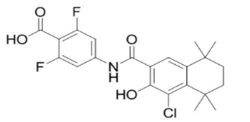		

AGN195183		No activity	RARα agonist
BMS753	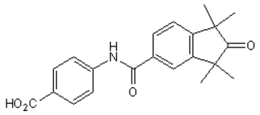	No activity	RARα agonist

*IC_{50} was determined by plaque reduction assay against MERS-CoV. This table not only provides information to facilitate structure-activity analysis of the compound, but also indicates that the RAR-α signaling pathway does not mediate the antiviral activity of AM580.

Supplementary Table 2. Primers used in this study.

Name	Sequence
ACC-F	GAGGGCTAGGTCTTTCTGGAAG
ACC-R	CCACAGTGAAATCTCGTTGAGA
FAS-F	AGCTGCCAGAGTCGGAGAAC
FAS-R	TGTAGCCCACGAGTGTCTCG
ADRP-F	GGGATCCCTGTCTACCAAGC
ADRP-R	AGATGTCGCCTGCCATCACC
CPT1a-F	TGAGCGACTGGTGGGAGGAG
CPT1a-R	GAGCCAGACCTTGAAGTAGCG
HADHα-F	GCTAGACCGAGGACAGCAAC
HADHα-R	CCTGCTTGAGACCAACTGCT
UCP2-F	CACCAAGGGCTCTGAGCATG
UCP2-R	TCTACAGGGGAGGCGATGAC
ACOX-F	TCCTGCCCACCTTGCTICAC
ACOX-R	TTGGGGCCGATGTCACCAAC
BOX-F	GGGCATTCCACATCCGGTTG
BOX-R	TGGCTCCTGAGCAGATCAGC
CYP2E1-F	ATGTCTGCCCTCGGAGTGA
CYP2E1-R	GATGTCCTTCCAGGTAGGTCC
CYP4A11-F	AGGAGCTCCAACAGGACCAG
CYP4A11-R	CCTGATGGCTGAAGGCACAC
PPARα-F	CCAGTATTTAGGAAGCTGTCCTG
PPARα-R	CGTTGTGTGACATCCCGACAG
SOD-F	AGGCCGTGTGCGTGCTGAAG
SOD-R	CACCTTTGCCCAAGTCATCTGC
Catalase-F	CCTTTCTGTTGAAGATGCGGCG
Catalase-R	GGCGGTGAGTGTCAGGATAG
GSS-F	AGAACGCTGCCTTCCTGGAG
GSS-R	CAGTAGCACCAGAGCATTGGG
DGAT1-F	GGCATCCTGAACTGGTGTGTG
DGAT1-R	GAGCTTGAGGAAGAGGATGGTG
PPARγ-F	GAACAGATCCAGTGGTTGCAG
PPARγ-R	GGCATTATGAGACATCCCCAC
HSL-F	TACCGCAGCCTAGTGCACAC
HSL-R	AGATGGTCTGCAGGAATGGC
HMGCS1-F	GAGGGCTTCGTGGGACACATA
HMGCS1-R	GCCACTGGGCATGGATCTTT
HMGCR-F	GGGAACCTCGGCCTAATGAA
HMGCR-R	CACCACGCTCATGAGTTTCCA
MVK-F	CCTTGTGGCTGGCGTCAGAAA
MVK-R	CGAGGGCATTCAGATGGTGCT
PMVK-F	GCCTTTCTCTCCGCGTGTCT

PMVK-R	GGAGCGGCAACAAGGAACA
SQLE-F	GCGTGCTTGGCTCTGCTTT
SQLE-R	CCTGGGCATCAAGACCTTCCA
LSS-F	GCACTGGACGGGTGATTATGGT
LSS-R	CGCAGGTACCGCACAATCTCTT
CYP51A1-F	CAGGGATTGATCCGCCTCTTCA
CYP51A1-R	CACAGAATGGGGCGGGATGTT
DHCR7-F	GGGGCCGGTTCAAGAAGGAAA
DHCR7-R	GCCCTTGAGATGCGGTTCTGT
LDLR-F	GCAGTGGGCGACAGATGTGAA
LDLR-R	GCACGTCTCCTGGGACTCATCA
SQS-F	CAAGAGGTTTGGAGCAGGTATG
SQS-R	ACTGCACGGCCAAGTCAATA
ACL-F	CCTCGAGATCAATCCCCTTGTA
ACL-R	CGATGTCACCCCACTTCACTTT
SCD-F	TGCTGCCCACCTCTTCGGATAT
SCD-R	TAGTTGTGGAAGCCCTCACCCA
TNF-α-F	CCCAGGGACCTCTCTCTAATC
TNF-α-R	ATGGCTACAGGCTTGTCACT
IL-1β-F	GCAGCCATGGCAGAAGTACCTGA
IL-1β-R	CCAGAGGGCAGAGGTCCAGGTC
IL-6-F	CACAGAGGATACCACTCCCAACA
IL-6-R	TCCACGATTTCCCAGAGAACA
IL-8-F	ATACTCCAAACCTTTCCACCC
IL-8-R	TCTGCACCCAGTTTTCCTTG
IFN-α-F	TGACCTCAAAGCCTGTGTGATG
IFN-α-R	AAGTATTTCCTCACAGCCAGCAG
IFN-β-F	GACGCCGCATTGACCATCTA
IFN-β-R	CCTTAGGATTTCCACTCTGACT
IFN-γ-F	CTTTAAAGATGACCAGAGCATCCA
IFN-γ-R	ATCTCGTTTCTTTTTGTTGCTATTGA
MERS-CoV-NP-F	CAAAACCTTCCCTAAGAAGGAAAAG
MERS-CoV-NP-R	GCTCCTTTGGAGGTTCAGACAT
Influenza-HA-F	CAATAAGACCCAAAGTGAGGG
Influenza-HA-R	AATCGTGGACTGGTGTATCTG
EV-A71-VP1-F	GAGAGTTCTATAGGGGACAGT
EV-A71-VP1-R	AGCTGTGCTATGTGAATTAGGAA
AdV-5-hexon-F	AGTGGKCDTACATGCACATC
AdV-5-hexon-R	CGGGCRAAYTGCACSAG
hGAPDH-F	ATTCCACCCATGGCAAATTC
hGAPDH-R	CGCTCCTGGAAGATGGTGAT
mGAPDH-F	AAGGTCATCCCAGAGCTGAA
mGAPDH-R	CTGCTTCACCACCTTCTTGA
