# Supplementary material for: SREBP-dependent lipidomic reprogramming as a broad-spectrum antiviral target
Source: Nat Commun. 2019 Jan 10;10:120. doi: 10.1038/s41467-018-08015-x (PMC6328544; doi:10.1038/s41467-018-08015-x)
Supplement: Supplementary file 3 — Description of Additional Supplementary Files [file 41467_2018_8015_MOESM3_ESM.pdf]

Description of Additional Supplementary Files

File Name: Supplementary Data 1
Description: ‘Identified lipids that were significantly changed after MERS-CoV infection with or without AM580 treatment
