# Supplementary material for: SREBP-dependent lipidomic reprogramming as a broad-spectrum antiviral target
Source: Nat Commun. 2019 Jan 10;10:120. doi: 10.1038/s41467-018-08015-x (PMC6328544; doi:10.1038/s41467-018-08015-x)
Supplement: Supplementary file 4 — Supplementary Data 1 [file 41467_2018_8015_MOESM4_ESM.docx]

**Identified lipids in the lipidomic analysis that were significantly changed after MERS-CoV infection with or without AM580 treatment ^a^**

| **Lipid name ^b^** | **Detection mode ^c^** | **Retention time (min)** | **Accurate mass** | **Identification confidence ^d^** | **T-test**  **MERS-CoV /Mock** | **T-test**  **MERS-CoV with AM580/ MERS-CoV** | **Fold change (MERS-CoV/**  **Mock)** | **Fold change (MERS-CoV with AM580/MERS-CoV)** |
| --- | --- | --- | --- | --- | --- | --- | --- | --- |
| Cer(d18:1/16:0) | Neg | 27.2 | 536.504 | STD | 0.0004 | 0.0088 | 0.45 | 1.94 |
| PC(16:0/0:0) | Neg | 12.7 | 554.345 | STD | 0.0002 | 0.2823 | 0.47 | 1.28 |
| FA(16:1) | Neg | 15 | 253.218 | MS2 | 0.0005 | 0.0489 | 0.42 | 1.81 |
| FA(18:1) | Neg | 12.75 | 281.2494 | MS2 | 0.0042 | 0.1199 | 0.69 | 1.18 |
| FA(20:2)_B | Neg | 17.97 | 307.2643 | MS2 | 0.0173 | 0.1069 | 0.43 | 1.93 |
| FA(20:1)_B | Neg | 19.57 | 309.2805 | MS2 | 0.0002 | 0.0944 | 0.38 | 1.75 |
| PC(P-16:0) | Pos | 11.68 | 494.3147 | MS2 | 0.0004 | 0.1081 | 0.42 | 1.69 |
| PC(28:0)_A | Pos | 22.93 | 678.4881 | MS2 | 0.0352 | 0.0040 | 0.71 | 2.36 |
| PC(28:0)_B | Pos | 23.51 | 678.486 | MS2 | 0.0027 | 0.0004 | 0.53 | 2.85 |
| PC(32:2) | Pos | 23.62 | 730.5255 | MS2 | 0.0081 | 0.0021 | 0.47 | 1.96 |
| PC(34:3) | Pos | 23.95 | 756.5382 | MS2 | 0.0026 | 0.0000 | 0.21 | 4.07 |
| PE(18:1/0:0) | Neg | 12.98 | 478.2941 | MS2 | 0.0011 | 0.0000 | 0.46 | 2.16 |
| PC(38:5)_A | Pos | 22.51 | 808.5608 | MS2 | 0.0462 | 0.0407 | 1.40 | 0.80 |
| PC(38:5)_B | Pos | 16.76 | 808.5613 | MS2 | 0.0295 | 0.1915 | 20.56 | 0.41 |
| PC(38:2)_A | Pos | 26 | 814.6099 | MS2 | 0.0322 | 0.0367 | 4.51 | 0.25 |
| PC(14:0/0:0) | Neg | 11.29 | 526.312 | MS2 | 0.0009 | 0.0303 | 0.33 | 2.44 |
| PC(0:0/16:1) | Neg | 11.44 | 552.3281 | MS2 | 0.0037 | 0.0048 | 0.39 | 2.06 |
| PC(16:1/0:0) | Neg | 11.69 | 552.3292 | MS2 | 0.0004 | 0.0168 | 0.34 | 2.34 |
| PC(18:1/0:0) | Neg | 13 | 580.3613 | MS2 | 0.0000 | 0.0238 | 0.38 | 1.71 |
| PC(0:0/18:1) | Neg | 12.77 | 580.3616 | MS2 | 0.0001 | 0.0002 | 0.48 | 1.78 |
| PC(18:0/0:0) | Neg | 14.13 | 582.3763 | MS2 | 0.0001 | 0.0681 | 0.38 | 1.80 |
| PC(28:0) | Neg | 22.86 | 736.5103 | MS2 | 0.0416 | 0.0634 | 0.47 | 2.75 |
| FA(16:0) | Neg | 12.71 | 255.2335 | MS | 0.0001 | 0.2137 | 0.51 | 1.16 |
| FA(20:2)_A | Neg | 17.6 | 307.2646 | MS | 0.0185 | 0.3931 | 0.35 | 1.97 |
| FA(20:1)_A | Neg | 14.07 | 309.2799 | MS | 0.0068 | 0.1242 | 0.52 | 1.18 |
| PC(16:1) | Pos | 23.28 | 508.2909 | MS | 0.0163 | 0.6198 | 2.28 | 0.87 |
| PC(P-18:0) | Pos | 23.28 | 522.3377 | MS | 0.0006 | 0.0322 | 4.20 | 0.48 |
| PC(P-20:0) | Pos | 14.35 | 550.373 | MS | 0.0481 | 0.9935 | 0.49 | 1.00 |
| FA(22:4) | Neg | 12.87 | 331.2625 | MS | 0.0380 | 0.5430 | 0.65 | 1.15 |
| SM(26:0) | Pos | 19.24 | 593.4659 | MS | 0.0005 | 0.0376 | 0.35 | 2.16 |
| FA(22:2) | Neg | 20.07 | 335.2958 | MS | 0.0028 | 0.2187 | 0.15 | 3.57 |
| FA(22:1)_A | Neg | 22.39 | 337.3113 | MS | 0.0009 | 0.1375 | 0.41 | 1.48 |
| FA(22:1)_B | Neg | 21.97 | 337.3116 | MS | 0.0065 | 0.1062 | 0.33 | 1.97 |
| FAHFA(20:0) | Neg | 16.89 | 341.2693 | MS | 0.0455 | 0.2274 | 0.12 | 0.23 |
| SM(34:0) | Pos | 24.49 | 705.5915 | MS | 0.0104 | 0.3057 | 0.30 | 1.26 |
| PC(30:0)_A | Pos | 16.23 | 706.545 | MS | 0.0092 | 0.9341 | 3.61 | 1.02 |
| PC(30:0)_B | Pos | 21.55 | 706.5492 | MS | 0.0002 | 0.3777 | 5.42 | 0.78 |
| PC(P-32:0) | Pos | 23.59 | 718.5493 | MS | 0.0139 | 0.5758 | 2.29 | 0.89 |
| PE(P-36:4) | Pos | 13.32 | 724.5114 | MS | 0.0128 | 0.0987 | 2.54 | 0.47 |
| PC(32:1) | Pos | 26.07 | 732.5308 | MS | 0.0289 | 0.0067 | 0.64 | 1.44 |
| PC(32:0) | Pos | 16.3 | 734.5511 | MS | 0.0290 | 0.9533 | 6.64 | 1.03 |
| PC(34:6) | Pos | 17.45 | 750.5246 | MS | 0.0210 | 0.3249 | 0.40 | 1.72 |
| PC(34:2) | Pos | 26.62 | 758.5468 | MS | 0.0396 | 0.0334 | 0.68 | 1.20 |
| PC(34:1)_A | Pos | 28.8 | 760.5649 | MS | 0.0297 | 0.0842 | 1.68 | 0.67 |
| PC(34:1)_B | Pos | 16.33 | 760.5678 | MS | 0.0486 | 0.4337 | 3.43 | 0.72 |
| PC(P-36:1) | Pos | 24.25 | 772.5981 | MS | 0.0130 | 0.1931 | 2.32 | 0.68 |
| PC(36:6) | Pos | 23.82 | 778.5523 | MS | 0.0064 | 0.3053 | 0.62 | 1.14 |
| PC(36:4)_A | Pos | 19.25 | 782.5464 | MS | 0.0095 | 0.0150 | 2.35 | 0.64 |
| PC(36:4)_B | Pos | 16.75 | 782.5482 | MS | 0.0209 | 0.3661 | 14.89 | 0.57 |
| PC(P-38:5) | Pos | 23.82 | 792.562 | MS | 0.0001 | 0.0052 | 7.46 | 0.29 |
| PC(P-38:3) | Pos | 23.26 | 796.5855 | MS | 0.0234 | 0.3209 | 2.32 | 0.78 |
| PC(38:4) | Pos | 26.52 | 810.572 | MS | 0.0289 | 0.0075 | 1.95 | 0.68 |
| PE(20:2/0:0) | Neg | 13.34 | 504.308 | MS | 0.0327 | 0.4094 | 0.79 | 1.07 |
| PE(P-20:0) | Neg | 12.99 | 506.3246 | MS | 0.0000 | 0.0115 | 0.54 | 1.38 |
| PC(38:2)_B | Pos | 27.49 | 814.6108 | MS | 0.0373 | 0.0059 | 1.80 | 0.67 |
| PG(18:1/0:0)_A | Neg | 16.41 | 509.2873 | MS | 0.0032 | 0.0945 | 0.25 | 4.11 |
| PG(18:1/0:0)_B | Neg | 16.65 | 509.2881 | MS | 0.0374 | 0.0047 | 0.15 | 3.67 |
| PI(16:0/0:0) | Neg | 15.05 | 571.2853 | MS | 0.0436 | 0.4586 | 0.59 | 1.13 |
| FAHFA(38:4) | Neg | 26.22 | 585.4829 | MS | 0.0361 | 0.3866 | 0.41 | 1.53 |
| PI(18:1/0:0) | Neg | 15.29 | 597.3032 | MS | 0.0304 | 0.2068 | 0.20 | 2.11 |
| PI(18:0/0:0) | Neg | 18.74 | 599.3184 | MS | 0.0493 | 0.0414 | 0.40 | 3.15 |
| PC(20:1/0:0) | Neg | 14.32 | 608.39 | MS | 0.0021 | 0.0554 | 0.29 | 2.09 |
| PA(30:1) | Neg | 16.72 | 617.4168 | MS | 0.0440 | 0.8185 | 0.11 | 1.05 |
| PA(36:2) | Neg | 24.33 | 699.496 | MS | 0.0185 | 0.0383 | 0.39 | 2.17 |
| PE(34:2) | Neg | 12.21 | 714.5091 | MS | 0.0006 | 0.0003 | 1.98 | 0.62 |
| MGDG(30:1) | Neg | 21.36 | 759.5367 | MS | 0.0487 | 0.1644 | 0.47 | 1.72 |
| PS(34:1) | Neg | 26.24 | 760.5177 | MS | 0.0000 | 0.9843 | 0.38 | 1.00 |
| PE(38:5) | Neg | 26.96 | 764.519 | MS | 0.0277 | 0.5962 | 0.33 | 1.51 |

**^a^** Calu-3 cells were mock infected or infected with MERS-CoV at 2 MOI and treated in the presence or absence of AM580. At 24 hpi, cells were collected for lipidomic analysis as described in the main text.

**^b^** A & B represented the lipids are isomer that has the same accurate mass but with a different retention time.

**^c^** Pos and Neg represented positive mode and negative mode respectively.

**^d^** STD represented the lipids were confirmed with authentic standards; MS2 represented the lipids were annotated and matched the fragmentation pattern with database; MS represented the lipids that were putatively annotated and matched the accurate mass with MS-DIAL internal database and website database.
