# Supplementary material for: SREBP-dependent lipidomic reprogramming as a broad-spectrum antiviral target
Source: Nat Commun. 2019 Jan 10;10:120. doi: 10.1038/s41467-018-08015-x (PMC6328544; doi:10.1038/s41467-018-08015-x)
Supplement: Supplementary file 5 — Reporting Summary [file 41467_2018_8015_MOESM5_ESM.pdf]

nature research

Corresponding author(s): Kwok-yung Yuen

Reporting Summary

Nature Research wishes to improve the reproducibility of the work that we publish. This form provides structure for consistency and transparency in reporting. For further information on Nature Research policies, see Authors & Referees and the Editorial Policy Checklist.

Statistical parameters

When statistical analyses are reported, confirm that the following items are present in the relevant location (e.g. figure legend, table legend, main text, or Methods section).

n/a	Confirmed	
☐	☒	The exact sample size (*n*) for each experimental group/condition, given as a discrete number and unit of measurement
☐	☒	An indication of whether measurements were taken from distinct samples or whether the same sample was measured repeatedly
☐	☒	The statistical test(s) used AND whether they are one- or two-sided<br>*Only common tests should be described solely by name; describe more complex techniques in the Methods section.*
☒	☐	A description of all covariates tested
☒	☐	A description of any assumptions or corrections, such as tests of normality and adjustment for multiple comparisons
☐	☒	A full description of the statistics including central tendency (e.g. means) or other basic estimates (e.g. regression coefficient) AND variation (e.g. standard deviation) or associated estimates of uncertainty (e.g. confidence intervals)
☒	☐	For null hypothesis testing, the test statistic (e.g. *F*, *t*, *r*) with confidence intervals, effect sizes, degrees of freedom and *P* value noted<br>*Give P values as exact values whenever suitable.*
☒	☐	For Bayesian analysis, information on the choice of priors and Markov chain Monte Carlo settings
☒	☐	For hierarchical and complex designs, identification of the appropriate level for tests and full reporting of outcomes
☒	☐	Estimates of effect sizes (e.g. Cohen's *d*, Pearson's *r*), indicating how they were calculated
☐	☒	Clearly defined error bars<br>*State explicitly what error bars represent (e.g. SD, SE, CI)*

Our web collection on statistics for biologists may be useful.

Software and code

Policy information about availability of computer code

Data collection	Acquisition of the raw lipidomic data was performed using MassLynx software version 4.1 (Waters Corp., MA, USA) and these raw data were firstly converted into the Analysis Base File (ABF) format. Transcriptomic clean reads are mapped to reference using HISAT and Bowtie2 tools.
Data analysis	Statistical analyses were conducted using GraphPad Prism (version 7.0).

For manuscripts utilizing custom algorithms or software that are central to the research but not yet described in published literature, software must be made available to editors/reviewers upon request. We strongly encourage code deposition in a community repository (e.g. GitHub). See the Nature Research guidelines for submitting code & software for further information.

Data

Policy information about availability of data

All manuscripts must include a data availability statement. This statement should provide the following information, where applicable:

- Accession codes, unique identifiers, or web links for publicly available datasets
- A list of figures that have associated raw data
- A description of any restrictions on data availability

All relevant data are available from the authors upon request. Lipidomics data have been deposited in MetaboLights under accession code MTBLS762. Transcriptomic data is available in GEO data repository under accession code GSE122876

Field-specific reporting

Please select the best fit for your research. If you are not sure, read the appropriate sections before making your selection.

☒ Life sciences ☐ Behavioural & social sciences ☐ Ecological, evolutionary & environmental sciences

For a reference copy of the document with all sections, see nature.com/authors/policies/ReportingSummary-flat.pdf

Life sciences study design

All studies must disclose on these points even when the disclosure is negative.

Sample size	The antiviral efficacy of the selected drug AM580 in mouse models has never been reported, thus we used 10-20 mice per group to improve data credibility.
Data exclusions	No data were excluded from the analyses.
Replication	All in vitro experiments was performed in triplicate and replicated twice for confirmation.
Randomization	Mice were randomly allocated to experimental groups.
Blinding	The experiments were not blinded.

Reporting for specific materials, systems and methods

Materials & experimental systems

n/a	Involved in the study	
☒	☐	Unique biological materials
☐	☒	Antibodies
☐	☒	Eukaryotic cell lines
☒	☐	Palaeontology
☐	☒	Animals and other organisms
☐	☒	Human research participants

Methods

n/a	Involved in the study	
☒	☐	ChIP-seq
☐	☒	Flow cytometry
☒	☐	MRI-based neuroimaging

Antibodies

Antibodies used	Rabbit-anti-RAR-α, Abcam Cat# ab41934; Mouse-anti-SREBP1 (A-4), Santa Santa Cruz Biotechnology Cat#sc-365513; Rabbit-anti-SREBP2, Proteintech, Cat# 14508-1-AP; Monoclonal ANTI-FLAG® M2 antibody, Sigma-Aldrich Cat#F3165; Alexa Fluor 488 goat anti-guinea pig IgG (H+L) antibody, Molecular Probes Cat# A-11073; Rabbit- anti-Influenza A virus H1N1 HA (Hemagglutinin) antibody, GeneTex Cat#GTX127357.
Validation	Validation statements are available on the manufacturer's website.

Eukaryotic cell lines

Policy information about cell lines

Cell line source(s)	ATCC and JCRB
Authentication	None of the cell lines used were authenticated.
Mycoplasma contamination	Cell lines were confirmed to be free of mycoplasma contamination by PlasmoTest (InvivoGen).
Commonly misidentified lines (See ICLAC register)	No cell lines used in this study is listed in ICLAC database.

Animals and other organisms

Policy information about studies involving animals; ARRIVE guidelines recommended for reporting animal research

Laboratory animals	Human dipeptidyl peptidase 4 (hDPP4) transgenic C57BL/6 mice (male and female, 6-8 weeks old) and BALB/c (female,6-8 weeks old) mice were used in this study.
Wild animals	This study did not involve wild animals.
Field-collected samples	This study did not involve field-collected samples.

Human research participants

Policy information about studies involving human research participants

Population characteristics	Intestinal organoids used in the study were derived from normal human colon from a surgical resection of a 6-year old female.
Recruitment	Informed consent was obtained before surgical resection. Because the intestinal organoids were used for antiviral evaluation with proper DMSO-treated control set, self-selection bias was minimized.

Flow Cytometry

Plots

Confirm that:

☒ The axis labels state the marker and fluorochrome used (e.g. CD4-FITC).

☒ The axis scales are clearly visible. Include numbers along axes only for bottom left plot of group (a 'group' is an analysis of identical markers).

☒ All plots are contour plots with outliers or pseudocolor plots.

☒ A numerical value for number of cells or percentage (with statistics) is provided.

Methodology

Sample preparation	All samples were detached with 10 mM EDTA and fixed in 4% paraformaldehyde. Cell permeabilization for intracellular staining was performed with 0.1% Triton X-100 in PBS.
Instrument	BD FACSCanto II flow cytometer
Software	The data were analyzed using FlowJo vX
Cell population abundance	This study did not involve cell population abundance
Gating strategy	Use the preliminary FSC/SSC gating to obtain the target cell population by remove the effect of dead cell and debris. The positive or negative cell population are defined by fluorescent signal compared with mock control.

☒ Tick this box to confirm that a figure exemplifying the gating strategy is provided in the Supplementary Information.
